# Positive and negative regulation of T cell responses by fibroblastic reticular cells within paracortical regions of lymph nodes

**DOI:** 10.3389/fimmu.2012.00285

**Published:** 2012-09-11

**Authors:** Stefanie Siegert, Sanjiv A. Luther

**Affiliations:** Department of Biochemistry, University of LausanneEpalinges, Switzerland

**Keywords:** lymph node stromal cells, immune tolerance, suppression, mesenchymal stem cells, T lymphocyte activation, fibroblastic reticular cell (FRC), inducible nitric oxide synthase, cyclooxygenase 2

## Abstract

Fibroblastic reticular cells (FRC) form the structural backbone of the T cell rich zones in secondary lymphoid organs (SLO), but also actively influence the adaptive immune response. They provide a guidance path for immigrating T lymphocytes and dendritic cells (DC) and are the main local source of the cytokines CCL19, CCL21, and IL-7, all of which are thought to positively regulate T cell homeostasis and T cell interactions with DC. Recently, FRC in lymph nodes (LN) were also described to negatively regulate T cell responses in two distinct ways. During homeostasis they express and present a range of peripheral tissue antigens, thereby participating in peripheral tolerance induction of self-reactive CD8^+^ T cells. During acute inflammation T cells responding to foreign antigens presented on DC very quickly release pro-inflammatory cytokines such as interferon γ. These cytokines are sensed by FRC which transiently produce nitric oxide (NO) gas dampening the proliferation of neighboring T cells in a non-cognate fashion. In summary, we propose a model in which FRC engage in a bidirectional crosstalk with both DC and T cells to increase the efficiency of the T cell response. However, during an acute response, FRC limit excessive expansion and inflammatory activity of antigen-specific T cells. This negative feedback loop may help to maintain tissue integrity and function during rapid organ growth.

Secondary lymphoid organs (SLO), such as lymph nodes (LN) and spleen, are essential for efficient initiation of adaptive immune responses. Their structure is thought to optimize antigen concentration and presentation to naive recirculating lymphocytes and allow for rapid selection and proliferation of antigen-specific T and B lymphocytes, as well as their differentiation into effector cells. To make these processes more efficient SLO are compartmentalized into functionally distinct microenvironments: the B zone (follicle or outer cortex), T zone (LN paracortex or splenic periarteriolar lymphoid sheath), and entry and exit zones. Three-dimensional networks of specialized radio-resistant fibroblasts (CD45- Ter119- CD31-) are responsible for generating and maintaining these B- and T-cell rich compartments. On the one hand, follicular dendritic cells (FDC; CD21/35+ FDC-M1/2+) inside B zones constitutively produce the chemokine CXCL13 to attract and retain B cells. On the other hand, fibroblastic reticular cells (FRC; CD21/35- gp38+) inside T zones constitutively express the chemokines CCL19 and CCL21 to attract and retain T cells and antigen-presenting dendritic cells (DC) [reviewed in (Cyster, 2005; Junt et al., 2008; Mueller and Germain, 2009; Turley et al., 2010)]. While our understanding of the origin and differentiation of these fibroblast subsets is still very limited (Cyster et al., 2000; Koning and Mebius, 2011), much progress has been made in defining novel roles of FRC in adaptive immunity.

Lymphocytes constantly recirculate between SLO. Once inside the SLO they continue migrating using a random walk to search their cognate antigen. T lymphocytes use the FRC network as physical guidance during this migration. B cells often move first across the T zone by following the FRC network before switching to the connecting FDC network when entering the B zone (Bajenoff et al., 2006). Finally, immigrating and tissue-resident DC associate with the T zone FRC network as well in order to present antigens to recirculating T cells (Lindquist et al., 2004; Bajenoff et al., 2006). It is within this FRC-rich T zone microenvironment where DC and T cells interact to find rare antigen-specific T cells and where some key decisions are being taken: Is a T cell response or rather tolerance induced? If there is a T cell response induced, what will be the type and magnitude? Where will the effector T cells be directed (reviewed in Haring et al., 2006; Junt et al., 2008; Mora et al., 2008; Mueller and Germain, 2009)? In this review we will discuss the recent findings which suggest that T zone FRC are not simple scaffolding cells, but actively influence these critical decisions. A major focus will be on the surprising finding that FRC not only enhance but may also suppress adaptive immunity.

## FRC as positive regulators of the T cell response

**Figure 1:**
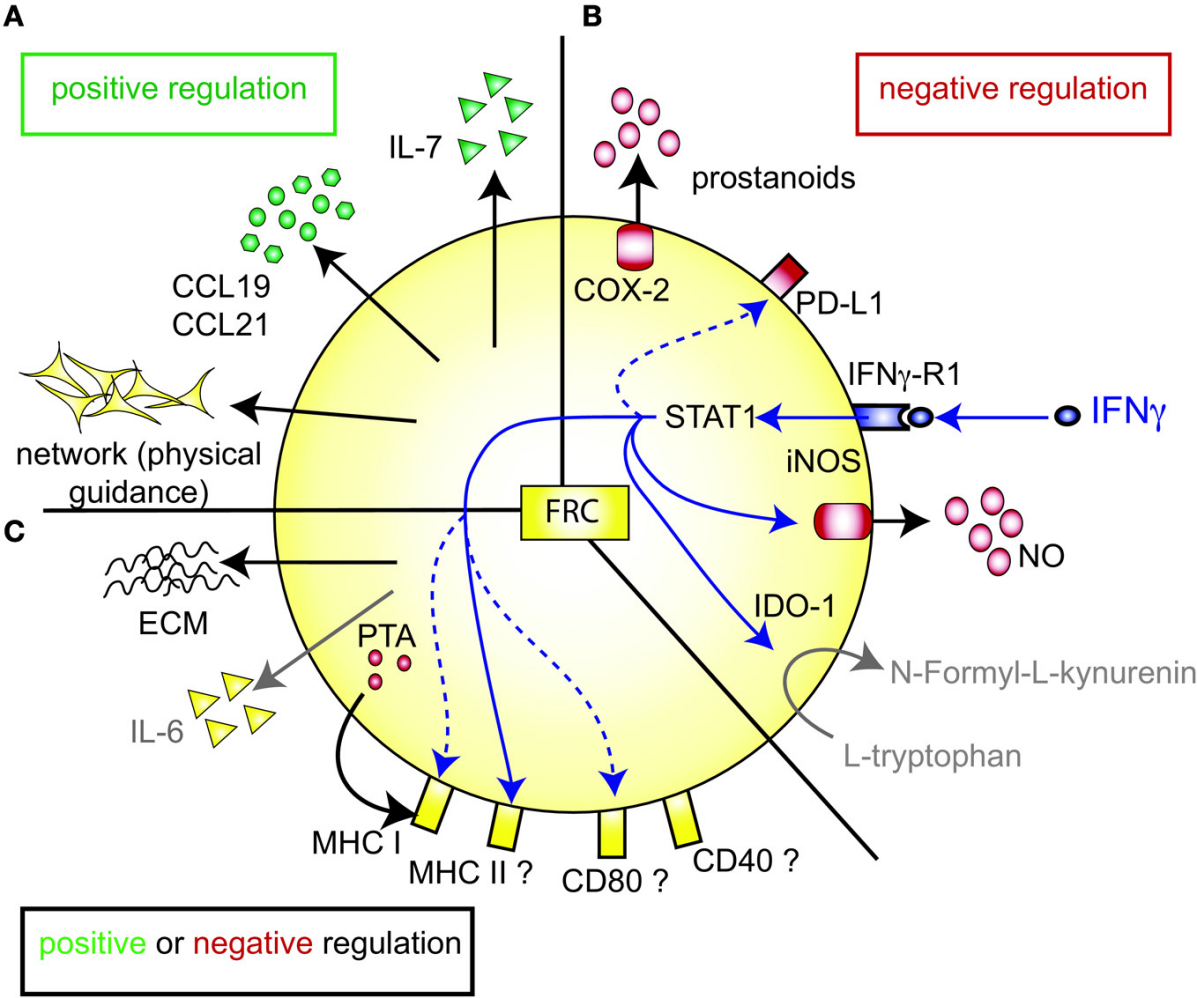
**FRC-expressed molecules that positively or negatively regulate the T cell response. (A)** FRC are thought to positively regulate T cell immunity in several ways. Throughout the T zone of SLO FRC form a three-dimensional network that serves as a scaffold for DC adhesion and T cell migration, thereby increasing DC-T interaction. In addition, FRC constitutively produce CCL19 and CCL21 that retain T cells in the T zone while increasing their motility. FRC also constitutively produce IL-7 that promotes T cell survival. CCL19, CCL21, and IL-7 may all serve as co-stimulatory signals in case of weak T cell receptor triggering. **(B)** FRC express several molecules that may negatively regulate the immune response: COX-2 and PD-L1 are constitutively expressed, while iNOS and IDO-1 are only expressed after induction by IFNγ. iNOS expression in FRC is also induced by other pro-inflammatory cytokines, such as TNFα, IL-1β and IFNα (not shown). PD-L1 expression on FRC is increased by LCMV infection, IFNγ or TLR3 stimulation (Mueller et al., 2007; Fletcher et al., 2010; Ng et al., 2012). While blocking iNOS and COX improves T cell proliferation in the presence of FRC, no impact of FRC-derived PD-L1, IDO-1, CD80/86, or IL-2 on T cell proliferation has been shown. It is unclear whether FRC can express L-arginase, but arginase inhibitors showed no effect *in vitro* (Khan et al., 2011; Lukacs-Kornek et al., 2011; Siegert et al., 2011). **(C)** FRC produce several factors which may regulate adaptive immunity in a positive or negative way. They secrete extracellular matrix (ECM) proteins some of which can bind IL-7 and CCL21 and thereby regulate cytokine availability and localization (Katakai et al., 2004b; Förster et al., 2008; Huang and Luther, 2012). ECM molecules may also directly modulate T cell and DC behavior. Microarray data suggest that FRC express constitutively IL-6, which is both anti- and pro-inflammatory depending on the context. FRC express MHC I and low levels of MHC II, CD80, and CD40. IFNγ or TLR3 stimulation enhances the expression of MHC I, MHC II and CD80. In addition, LCMV infection or inflammation evoked by LPS injection *in vivo* can induce MHC II expression on FRC. LN FRC express PTA, which are presented in the context of MHC I, leading to T cell proliferation followed possibly by clonal deletion of self-reactive T cells. Therefore, in certain settings FRC may act as APC.

T zone FRC are thought to orchestrate and enhance productive T-DC encounters in SLO by various means (Figure 1A). First, the FRC network acts as a 3D “road system” for T cells forcing them to pass by antigen-presenting DC attached to FRC, presumably enhancing the frequency of encounters between these two cell types (Katakai et al., 2004a; Lindquist et al., 2004; Sixt et al., 2005; Bajenoff et al., 2006; Mueller and Germain, 2009). This view was recently challenged by computer simulations of T cell trafficking within the LN FRC network which showed that mechanical guidance cues only enhance these encounters when FRC provoke T cell streams, or when FRC also provide motility factors such as the two CCR7 ligands CCL19 and CCL21 (Graw and Regoes, 2012). Second, FRC are the major constitutive source of CCL19 and CCL21, which bring T cells and DC into close proximity (Luther et al., 2000; Link et al., 2007). These chemokines also enhance the motility and survival of T cells (Stachowiak et al., 2006; Asperti-Boursin et al., 2007; Link et al., 2007; Okada and Cyster, 2007; Worbs et al., 2007) as well as the maturation, dendrite formation and antigen uptake by DC (Yanagawa and Onoé, 2002, 2003; Marsland et al., 2005). *In vitro* both CCL19 and CCL21 can act as co-stimulatory signals enhancing T cell priming, especially in settings with sub-optimal T cell stimulation (Flanagan et al., 2004; Friedman et al., 2006; Gollmer et al., 2009). *In vivo* the importance of CCR7 for mounting efficient T cell responses varies greatly depending on the model antigen or pathogen used [reviewed in (Förster et al., 2008; Junt et al., 2008)]. On the ligand side, CCL19 was shown by two laboratories to be dispensable for T cell activation *in vivo* (Saeki et al., 1999; Britschgi et al., 2010), with one report claiming the opposite (Robbiani et al., 2000). Currently, little is known about the *in vivo* role of FRC-derived CCL21, however, the lack of CCL21 expression in HEV of human LN suggests a key role for it in lymphocyte transmigration across HEV (Carlsen et al., 2005). Third, FRC are the major source of constitutive IL-7 expression in LN and thereby regulate the fitness, survival and homeostasis of naive recirculating T cells (Link et al., 2007; Hara et al., 2012; Huang and Luther, 2012). Adding or blocking IL-7 *in vitro* showed little effect on T cell receptor (TCR)-dependant T cell activation besides the improved viability of T cells and DC in presence of IL-7. However, IL-7 appears to be important *in vivo* for effective interactions between DC and T cells by enhancing TCR signaling and boosting primary antigen-specific T cell expansion (Saini et al., 2009; Mackall et al., 2011; Pellegrini et al., 2011; Huang and Luther, 2012). Administration of IL-7 *in vivo* also augments the effector function and memory formation of T cells (Pellegrini et al., 2011). Not surprisingly, IL-7 is regarded as an attractive adjuvant, which is currently being investigated in several clinical trials (Mackall et al., 2011; Huang and Luther, 2012). In conclusion, LN FRC positively regulate T cell migration and homeostasis. However, the evidence that FRC also augment T cell responses, namely T cell priming as well as effector and memory differentiation, is weaker and more indirect as it is often based on *in vitro* assays or on recombinant proteins expressed non-exclusively by FRC (Figure 1A).

## FRC as negative regulators of the T cell response

Paradoxically, recent studies have proposed that FRC also negatively regulate T cells, both during homeostasis and immune responses. On one hand, FRC induce T cell tolerance via self-antigen expression, on the other hand they can impair T cell responses to foreign antigens by expressing suppressive factors, either directly inhibiting T cell expansion or lowering the immunogenicity of DC (Figures 1B,C) (Fletcher et al., 2010; Turley et al., 2010; Khan et al., 2011; Lukacs-Kornek et al., 2011; Siegert et al., 2011).

**Figure 2:**
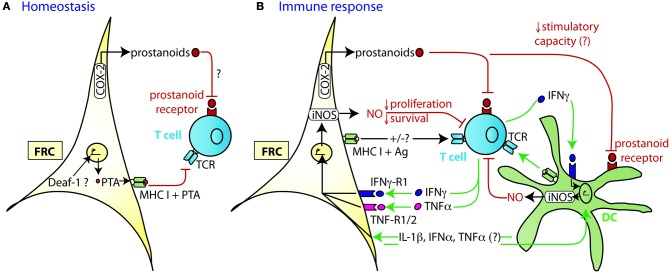
**Mechanism of TRC-mediated negative regulation of T cell expansion. (A)** During homeostasis LN FRC express self-antigens (PTA), possibly in a Deaf-1 dependent fashion. PTA presented in the context of MHC I may lead to peripheral tolerance induction via clonal deletion of self-reactive T cells. *Cox-2* is constitutively expressed in FRC, presumably leading to the production of PGE_2_ or other prostanoids that may attenuate T cell priming or proliferation. **(B)** During immune response DC capture antigen (Ag) in the periphery, home to the T zone of SLO and present peptides of foreign antigens to recirculating T cells. Within the first 20 h upon TCR triggering, T cells produce a first wave of IFNγ and TNFα cytokines, before entering cell cycle. In case of a very strong immune response, the high level of pro-inflammatory cytokines induces transient iNOS expression in the neighboring FRC and DC. The resulting NO release then acts as a negative feedback loop slowing down T cell proliferation over the following days presumably to ensure that the strong T cell response does not endanger tissue integrity. High concentrations of NO may also induce T cell apoptosis. The attenuating effect by FRC can occur in trans, without cognate interaction with T cells. Constitutive expression of COX-2 dependent factors, such as PGE_2_, may contribute to this attenuation of T cell expansion, by acting directly or indirectly (via DC) on the proliferating T cells. Not only is there crosstalk between FRC and T cells, but also between FRC and DC which leads to a much stronger release of NO by these two cell types if IFNγ is present. While the factors exchanged between FRC and DC have not yet been identified, the may include IL-1β, IFNα, and TNFα, all of which can trigger iNOS expression.

Similar to thymic epithelial cells (TEC), FRC from LN were shown to constitutively express multiple peripheral tissue restricted antigens (PTA), including known autoimmune targets (Figures 1C, 2A) (Fletcher et al., 2010; Turley et al., 2010). They share this feature with other LN stromal cells, such as lymphatic and blood endothelial cells (LEC and BEC) (Cohen et al., 2010; Fletcher et al., 2010), although the PTA are only partially overlapping. However, the best characterized regulator of promiscuous gene expression, Autoimmune regulator (Aire), was found only in a rare EpCAM-expressing cell type in the outer T zone of LN (Gardner et al., 2008), while Aire is poorly expressed in FRC, LEC, and BEC (Fletcher et al., 2010) (our unpublished observation). Another potential positive regulator of promiscuous gene expression, deformed epidermal autoregulatory factor 1 (Deaf1), was observed to be strongly expressed in FRC as well as other LN stromal cell types (Fletcher et al., 2010; Turley et al., 2010). What is the functional consequence of PTA expression by FRC? Turley and colleagues reported that in transgenic mice expressing a truncated ovalbumin (OVA), transferred OVA-specific CD8+ T cells in peripheral LN (pLN) proliferated followed by their partial deletion, even when MHC I was absent on hematopoietic cells. Surprisingly, the intestinal fatty acid binding promoter used to drive transgene expression led to OVA-peptide expression on MHC I selectively in gp38^+^ CD31^−^ FRC of pLN along with some expression of CD40 and CD80 (Lee et al., 2007; Fletcher et al., 2010). Based on these findings it was proposed that FRC can induce peripheral T cell tolerance but more direct evidence is needed to strengthen this notion. When these transgenic mice were bred to mice deficient in inducible nitric oxide synthase (iNOS, NOS2), OVA-specific T cell expansion was enhanced (Lukacs-Kornek et al., 2011). As FRC and LEC but not DC or macrophages expressed iNOS in this system it suggests stromal cells use nitric oxide (NO) to limit T cell expansion and possibly tolerance induction. While FRC can express MHC class II under certain conditions, e.g., upon IFNγ stimulation (Lee et al., 2007; Ng et al., 2012), its functional significance for tolerance and immunity has remained unexplored. Also unclear is why the typically inducible cyclooxygenase-2 (COX-2) enzyme is constitutively expressed by FRC in naive LN (Siegert et al., 2011). COX-2 is the rate-limiting enzyme for the generation of prostanoids, including prostaglandin E_2_ (PGE_2_), that can both enhance or suppress T cell immunity [reviewed in (Gualde and Harizi, 2004; Kalinski, 2012)]. It remains to be tested whether COX-2 participates in peripheral T cell tolerance induction. In conclusion, the existing evidence suggests that FRC and LEC assist DC in peripheral tolerance induction of self-reactive CD8+ T cells by expressing self-antigens and NO.

Besides their tolerance-promoting role as unconventional antigen presenting cells (APC), we and others recently provided evidence for the surprising capacity of FRC to attenuate the proliferation of CD4+ and CD8+ T cells in acute inflammation, independently of antigen presentation by FRC (Khan et al., 2011; Lukacs-Kornek et al., 2011; Siegert et al., 2011) (Figures 2A,B). FRC did not interfere with initial TCR signaling in primed T cells but with entry or progression in cell cycle and possibly with survival. The inhibitory effect by FRC was mainly mediated by iNOS dependent NO production, with iNOS being strongly induced in FRC by the synergistic action of IFNγ and TNFα. These two cytokines are produced by CD8^+^ T cells less than 24 h after priming and appear to be sensed by neighboring FRC (Figure 2B) (Khan et al., 2011; Lukacs-Kornek et al., 2011; Siegert et al., 2011), consistent with IFNγ having been detected over a wide area inside activated LN T zones (Beuneu et al., 2010; Perona-Wright et al., 2010). This early burst of cytokines before blastogenesis has previously been documented (Mempel et al., 2004; Beuneu et al., 2010) and induces crosstalk between these two cell types, leading to a negative feedback loop limiting T cell expansion in case of acute inflammation. NO is a highly reactive gas which is thought to be rapidly consumed by neighboring cells. NO or NO-derived reactive nitrogen species may negatively regulate T cells via nitrosylation of diverse amino acid residues, leading to down regulation of the TCR complex (Kasic et al., 2011). Furthermore, depletion of the iNOS substrate L-arginine may inhibit T cells, through down-regulation of CD3ζ (Rodriguez et al., 2002). Lastly, NO may directly block STAT5 phosphorylation thereby interfering with IL-2 signaling (Bingisser et al., 1998) [reviewed in Bogdan (2001), (2011)]. Notably, NO expression by FRC attenuated T cell proliferation without abrogating it (Khan et al., 2011; Lukacs-Kornek et al., 2011; Siegert et al., 2011). We therefore propose that FRC form micro-niches within the T zone where T cell proliferation is either enhanced or diminished, depending on the context or phase of immune response. Although IFNγ-stimulated FRC express other known suppressive molecules, such as programmed cell death ligand 1 (PD-L1) or Indolamin-2,3-dioxygenase (IDO) (Figure 1B), they do not seem to be implicated in the inhibition of T cell proliferation in the *in vitro* activation systems used (Khan et al., 2011; Lukacs-Kornek et al., 2011; Siegert et al., 2011). The function of PD-L1 on FRC may be protection from CD8+ T cell-mediated cytotoxicity, as in chronic LCMV infection (Mueller et al., 2007). A role for IDO in FRC remains to be established. As both PD-L1 and IDO have roles for regulatory T cells and suppressive myeloid cells, it would be interesting to address the function of FRC in the generation, maintenance or activation of these regulatory cells (Francisco et al., 2010; Le Blanc and Mougiakakos, 2012).

T cell expansion was also strongly attenuated *in vitro* when antigen-pulsed DC were used instead of T cell mitogens. In this setting, both iNOS and COX-2 were responsible for the strongly reduced T cell expansion. In comparison to proliferation, T cell effector function (killing, IFNγ expression) was less strongly affected (Siegert et al., 2011). Part of the attenuating effect on T cell expansion may be due to diminished DC immunogenicity. Overnight incubation of bone-marrow derived DC (BMDC) with FRC led to a 50% reduction of CD8+ T cell stimulation by these “FRC-conditioned” DC (Siegert et al., 2011). No differences in the expression of MHC I or CD80/86 by DC were observed suggesting alterations in the cytokine milieu as the most likely cause. PGE_2_ may be the culprit: While it stimulates immature DC in the periphery, it seems to be suppressive for mature DC in SLO and decreases their APC function (Gualde and Harizi, 2004). Interestingly, in presence of activated T cells crosstalk between DC and FRC strongly increases iNOS expression in both cell types. Similar to a previous report on rat lung fibroblasts (Lavnikova and Laskin, 1995), it is always only a fraction of FRC and DC which express iNOS, even when FRC clones or abundant IFNγ are used (Serbina et al., 2003; Siegert et al., 2011). It remains to be shown whether this is due to the cell cycle stage or a potential negative feedback loop preventing excessive iNOS activity. Currently, the signals exchanged between FRC and DC leading to iNOS expression is unclear. It seems likely that pro-inflammatory cytokines, like IL-1β and TNFα produced by activated DC are sensed by FRC leading to iNOS induction. Many cytokines were recently described to be produced by FRC (Fletcher et al., 2011; Malhotra et al., 2012), but the FRC-derived signals stimulating iNOS expression in DC remain to be identified. In contrast to these fairly rapid attenuating effects of FRC on DC, earlier studies have suggested that stromal cells can also have long-term effects on DC by promoting the development of regulatory DC which inhibit T cell responses. Adult splenic stromal cells co-cultured for 1 week with splenic c-kit^+^ precursor cells induced the development of CD11c^low^ CD45RB^+^ regulatory DC expressing IL-10 (Svensson et al., 2004). In another study, neonatal splenic stromal cells converted LPS-activated BMDC into NO-expressing regulatory DC, in a process dependent on cell-cell contact and TGFβ (Zhang et al., 2004). In both studies, the splenic stromal cells were only partially characterized and it is unclear whether these included FRC-like cells. These latter observations may be relevant for tissue development and chronic immune responses.

That FRC exert a suppressive effect on acute T cell expansion was best described in co-culture experiments, but *in vivo* experiments also support this notion. When wildtype (WT) mice that had previously received OVA-specific CD8^+^ T cells were infected with OVA expressing vesicular stomatitis virus (VSV-OVA), iNOS expression was observed only in a subset of FRC and, to a lesser extent, DC within draining LN (Siegert et al., 2011). This expression was detectable 24 h after infection, but not at later time points. However, the negative effects of iNOS on T cell expansion were clearly visible on day 4 and 8 suggesting NO either affects T cells only during the early activation phase or it permanently retards T cell proliferation due to longer lasting modifications in the target cells. Consistent with a negative role of iNOS *in vivo*, VSV-OVA infected iNOS^−/−^ mice showed an exaggerated expansion of antigen-specific T cells compared to WT mice (Siegert et al., 2011). When iNOS^−/−^ mice were immunized with OVA-loaded BMDC (Khan et al., 2011) a similar role for NO in restraining the T cell response was found. However, when the antigen was directly targeted to LN resident non-inflammatory DC using OVA-coupled anti-DEC205 antibodies, a decrease rather than an increase in antigen-specific T cell expansion was observed in iNOS-deficient mice (Khan et al., 2011). These findings support a model in which iNOS-mediated inhibition of T cell expansion is observed only in strong T_H1_-type immune responses characterized by high levels of IFNγ and TNFα. Consistent with this notion, T_H2_ cytokines did not induce iNOS in FRC and *in vitro* expansion of T_H2_ cells was not inhibited by FRC or iNOS (Niedbala et al., 1999; Khan et al., 2011). Currently it cannot be ruled out that other known iNOS sources, such as subsets of DC and macrophages, localize to LN T zones during T_H1_-type responses and contribute to these attenuating effects (Bogdan, 2001; Serbina et al., 2003; Siegert et al., 2011). Besides using NO within their lysosomes to kill phagocytosed microbes, both DC and macrophages (or myeloid-derived suppressor cells) can inhibit T cell proliferation *in vitro* by producing extracellular NO (Albina et al., 1991; Serbina et al., 2003; Zhang et al., 2004; Gabrilovich and Nagaraj, 2009; Siegert et al., 2011). In an attempt to better define the effect of iNOS expression in FRC versus DC, immune responses were investigated in irradiated iNOS^−/−^ mice receiving WT BM, but results were not conclusive as these mice showed unexpected defects in LN swelling, probably due to iNOS-dependent vascular defects induced by irradiation (Khan et al., 2011; Siegert et al., 2011). In the future, mice with cell type-specific deletion of iNOS are needed to firmly establish the relative roles of the various iNOS sources in the regulation of T cell expansion.

IFNγ stands out as a major regulator of FRC function: It strongly induces iNOS, IDO-1, PD-L1 as well as several molecules involved in antigen presentation (Figures 1B,C) (Mueller et al., 2007; Fletcher et al., 2010; Khan et al., 2011; Lukacs-Kornek et al., 2011; Siegert et al., 2011; Ng et al., 2012). Typically, IFNγ is regarded mostly as a pro-inflammatory cytokine because it enhances various aspects of the immune response, including the activation of macrophages, clearance of intracellular pathogens, up-regulation of MHC molecules or the promotion of T_H1_ responses. However, IFNγ also has immune-regulatory functions: It negatively modulates expression of tissue-destructive enzymes, diminishes the recruitment of inflammatory cells such as neutrophils, suppresses T_H17_ differentiation and positively regulates T_REG_ differentiation under certain circumstances [reviewed in (Kelchtermans et al., 2008; Hu and Ivashkiv, 2009)]. Consistent with this dual role, IFNγ can augment or suppress auto-immunity, depending on the context and timing. A prominent example is experimental autoimmune encephalomyelitis (EAE) (Kelchtermans et al., 2008; Hu and Ivashkiv, 2009). In one EAE mouse model autoimmune disease is enhanced in IFNγ-deficient mice as indicated by the increase of proliferating T cells in the spleen and the central nervous system (Chu et al., 2000). This finding may be related to the observation that T_H1_ cells limit their own expansion in the later phase of the immune response by expressing IFNγ (Feuerer et al., 2006; Haring et al., 2006). In autoimmune disease some of the protective effects mediated by IFNγ seem to be due to its capacity to induce iNOS expression. Support for this notion comes from the observation that self-reactivity and the associated pathology observed in experimental models for EAE or myasthenia gravis are enhanced in iNOS^−/−^ mice (Bogdan, 2001, 2011; Shi et al., 2001; Xiao et al., 2008). The protective effect of iNOS was also observed with mycobacteria that are a critical part of complete Freund's adjuvant (CFA). When rodents are immunized with CFA they often do not develop subsequent autoimmunity, a phenomenon termed “adjuvant immunotherapy.” Interestingly, Kahn and colleagues showed that this protective CFA effect depends on iNOS, as well as on IFNγ and TNFα which are the likely inducers of iNOS within lymphoid tissues (Kahn et al., 2001). Possibly, IFNγ-induced NO production in FRC or other host cells participates in the active down-regulation of T cell responses to limit tissue damage associated with acute inflammation or autoimmunity. In the future it will be of interest to test whether iNOS expression in myeloid cells, FRC or other cell types is critical for this protection.

## Is attenuation of T cell proliferation a feature of all mesenchymal cells?

The suppressive activity recently observed with FRC is reminiscent of the increasing number of reports showing that mesenchymal stem/stromal cells (MSC), as well as adult fibroblasts from non-lymphoid tissues, potently inhibit T cell proliferation, both *in vitro* and *in vivo*. Some reports also state that MSC reduce the cytotoxicity and IFNγ expression in effector T cells (Haniffa et al., 2007; Jones et al., 2007; Uccelli et al., 2008; Haniffa et al., 2009; Khan et al., 2011; Siegert et al., 2011). Various mechanisms of suppression have been described for MSC and non-lymphoid fibroblasts, as summarized in Table 1 (Nauta and Fibbe, 2007; Uccelli et al., 2008). It suggests that these suppressive pathways are either redundant, activated in a context-dependent way or simply due to differences in the assays used. Similar to FRC, IFNγ-dependant induction of iNOS in MSC appears to be a major inhibitory pathway (Nauta and Fibbe, 2007; Ren et al., 2008; Uccelli et al., 2008), but the production of suppressive COX-1/2-dependent prostanoids has also been reported for MSC (Nauta and Fibbe, 2007; Ren et al., 2008; Uccelli et al., 2008). The expression of several other inhibitory molecules observed in MSC may be conserved in FRC and should be the focus of future studies (Table 1). Again, reminiscent of FRC, MSC are able to present antigens in the context of MHC II, without it being a requirement for their inhibitory effect (Chan et al., 2006; Stagg et al., 2006; Uccelli et al., 2008). For antigen presentation by MSC the IFNγ level seems to be crucial: while low levels induce MHC II expression and APC function, both are lost with higher IFNγ concentrations (Chan et al., 2006). It remains to be tested whether APC function of FRC is regulated in a similar fashion. If yes, then this regulation would be opposite to that for iNOS expression. Importantly, MSC have proven to successfully suppress autoimmune disease and graft versus host disease (Gvhd) in mice and humans (Haniffa et al., 2007; Jones et al., 2007; Uccelli et al., 2008; Sato et al., 2010; Le Blanc and Mougiakakos, 2012). This year Canada approved MSC transfers as a preventive treatment for Gvhd during bone marrow transplantations (Prochymal®, www.osiris.com). While MSC can home both to SLO and inflammatory sites it is currently not clear where and when T cell inhibition takes place. In conclusion, most mesenchymal cell types share with FRC T cell suppressive characteristics, independent of their differentiation state. Interestingly, non-mesenchymal cells such as blood and lymphatic endothelial cells from LN also suppress T cell activation via an IFNγ/NO-dependant pathway (Khan et al., 2011; Lukacs-Kornek et al., 2011). In addition, epithelial cells from healthy kidney and skin or gut tumors also potently suppress T cell expansion, indicating that this characteristic is shared with many other cell types (Siegert et al., 2011).

**Table 1:** **Suppressive molecules known to be expressed in MSC versus their characterization in FRC**.

**Suppressive molecules expressed by MSC**	**Constitutive expression**				**References**	
	**MSC (human and murine)**		**FRC (murine)**		**MSC**	**FRC**
	**Effect on T cell activation**		**Expressed?**	**Effect on T cell activation**		
COX-2 (PGE_2_ ?)	Attenuated T cell proliferation, Impaired maturation of monocytes into DC		Yes	Attenuated CD8 T cell proliferation	4–7	1–3
HGF	Attenuated T cell proliferation		Yes	ND	4–7	2, 3
TGFβ	Attenuated T cell proliferation		Yes	No effect	4–7	1, 2, 3
IL-6	Impaired maturation of monocytes into DC		Yes	ND	4–7	2, 3
HO1	Attenuated T cell proliferation		Yes	ND	4–7	2, 3
Soluble HLA-G5 / Qa-2 or H2-Q8	Attenuated T cell proliferation and cytotoxicity		Yes	ND	4–7	2, 3
FasL	Induction of T cell apoptosis		No	ND	4–8	2, 3
	**Induced expression**					
	**Induced by**	**Effect on T cell activation**	**Induced by**	**Effect on T cell activation**		
iNOS	IFNγ	Attenuated T cell proliferation	IFNγ, ILß, TNFα, IFNα	Attenuated T cell proliferation (CD4 and CD8)	4–7	1, 9, 10
IDO-1/2	IFNγ	Attenuated T cell proliferation	IFNγ	No effect	4–7	1, 9
IL-10	T cell contact	Release of sHLA-G5	ND	No effect	4–7	1

MSC have been shown to interfere with the maturation and antigen presentation of DC and with T cell proliferation, both by cell-cell contact and various soluble factors. Similar observations have been obtained for FRC. FRC attenuate T cell proliferation mostly by NO production, but also lower the immunogenicity of DC in unknown ways. COX-1/2 dependent prostanoids produced by FRC may me responsible for this effect on DC. Interestingly, several candidates previously described in MSC appear to be expressed by FRC as well, as suggested by recent gene array results. Abbreviations: HGF, Hepatocyte Growth Factor; TGFβ Transforming Growth Factor beta; HO1, Heme Oxygenase-1; ND, not determined. References: (1) Siegert et al. (2011) (2) gene array data from Fletcher et al. (2011) (3) gene array data from Malhotra et al., 2012 for 2 and 3: NCBI GEO accession number GSE15907, immgen.org, (4) reviewed in Uccelli et al., 2008, (5) reviewed in Haniffa et al. (2009), (6) Sato et al. (2010) (7) reviewed in Nauta and Fibbe (2007), (8) Akiyama et al. (2012) (9) Lukacs-Kornek et al. (2011) (10) Khan et al. (2011).

## Why do non-hematopoietic cells contribute to immune regulation?

The emerging concept is that hematopoietic cells are not the sole regulators of T cell tolerance and T cell responses but that many tissue cells can contribute, including FRC within LN. At present we can only speculate on the reasons for this complex regulation. One possible explanation is that the immune system is a powerful but also dangerous weapon that needs to be tightly controlled at many levels and anatomical sites to allow efficient immune-mediated protection while avoiding immune-mediated pathology. During homeostasis the body may keep most tissues in a slightly immune-suppressed state to avoid the activation of low affinity T cells that recognize self-antigens or harmless antigens. During inflammation there may be an upper limit to the extent of expansion allowed for a given T cell population and a given organ. In this context, the structural cells may act as mechanical and chemical sensors of the ongoing T cell response in an inflamed organ and adjust population dynamics to ensure ordered tissue growth and maintenance of functional structures. If excessive inflammatory signals are present many tissue-specific resident cells, including fibroblasts inside SLO or non-lymphoid tissues, may be activated to put a brake on the immune response. A key function of fibroblasts is to maintain tissue integrity. In case of damage, they have repair functions with the aim of re-establishing homeostasis and full functionality. Both self-reactive and beneficial, but hyperactive, T cell responses are a serious threat for SLO and the body as a whole. Disruption of SLO structure is frequently associated with immunodeficiency and therefore to be avoided [reviewed in (Junt et al., 2008; Mueller and Germain, 2009)]. So, giving structural tissue cells, like FRC, some control over a strong ongoing T cell response appears to be a reasonable solution to proactively protect the body. Similar to the multiple cell types expressing self-antigens, and thereby shaping the peripheral T cell repertoire, several cell types—hematopoietic and non-hematopoietic—are likely to participate in the regulation of responses to foreign antigens and danger signals. It will be an important challenge to identify individual contributions, as well as coordination, between these various regulatory cells. Interestingly, many cell types of the immune system have a subset which suppresses or regulates adaptive immunity, including regulatory T and B cells, regulatory DC and alternatively activated M2 macrophages (Steinman et al., 2003; Lund and Randall, 2010; Murray and Wynn, 2011; Rudensky, 2011). Therefore, it may not be surprising that LN FRC turn out to be both positive and negative regulators of adaptive immunity. Similar to the other cell types, it remains to be established whether there is a dedicated FRC subset for immune suppression, or whether they are plastic and adopt an inhibitory activity, depending on the localization (e.g., suppressive micro-niches within the T zone), the activation status (e.g., cytokine milieu and cell-cell interactions modulating FRC) and the time (e.g., phase of the immune response). In fibroblasts and FRC some characteristics appear to stay imprinted and they correlate with the anatomical localization and the environment during cellular development (Chang et al., 2002; Hammerschmidt et al., 2008; Buettner et al., 2011; Molenaar et al., 2011). It will be a challenge for future investigations to assess in detail the complex effects FRC have on T cells, either in the naive, activated, or memory stage. These effects are likely to be strongly dependent on FRC density, functional specialization, environmental factors (positive and negative), and strength and type of T cell response. Many of these studies will rely on *in vitro* assays where physiological settings need to be mimicked as closely as possible. These findings will need confirmation from *in vivo* experiments. Mouse lines expressing Cre recombinase in an FRC-specific manner are now available (Kraman et al., 2010; Chai et al., 2011; Onder et al., 2011) and should help to selectively manipulate molecules in FRC.

While FRC-like networks have been described for other SLO, such as Peyer's patches and spleen (Katakai et al., 2008; Mueller and Germain, 2009), it remains to be established whether they are functionally similar to LN FRC. In the placenta, mucosal surfaces, skin and tumors, tissue fibroblasts could also contribute to immune regulation, in a way comparable to LN FRC. Tertiary lymphoid tissues (TLT), which often develop at ectopic sites in response to chronic inflammation, like diabetes or atherosclerosis, also contain various stromal cell types found in LN, including FRC-like cells expressing CCL19/21 and IL-7, both in mice and humans (Manzo et al., 2005; Gräbner et al., 2009; Peduto et al., 2009; Link et al., 2011; Perros et al., 2012). While these FRC-like cells seem to have features positively regulating adaptive immunity within TLT, it is currently unclear whether they also regulate it negatively. Given the prominent IFNγ and iNOS expression in many chronic inflammatory diseases, a contribution by fibroblastic cells appears likely. In case of TLT developing during autoimmune disease it would be of interest to test whether these stromal cells could be trained to suppress self-reactive T cell responses over prolonged periods of time. Alternatively, depleting these ectopic FRC or reducing their positive regulatory factors could be another promising avenue assuming TLT are critical for pathogenesis.

As LN FRC have both positive and negative effects on immune responses, they do not seem ideal candidates for a cellular therapy in autoimmunity. This is in contrast to MSC and skin fibroblasts, which are predominantly immunosuppressive. We postulate that during evolution, when LN developed in mammals, normal tissue fibroblasts were included to structure the LN (Boehm et al., 2012). In that process they may have kept their tissue-protective, immune-suppressive features, but have specialized further to include functions helping lymphocytes to survive (IL-7), to localize and migrate correctly (CCL19/21), to be silenced (PTA) or to be activated (conduits, IL-7, CCL19/21). The identification of the transcription factors, siRNA or epigenetic elements regulating the polarization of tissue fibroblasts should be an exciting field for future studies. Most importantly, future research may give clinicians the ability to modulate fibroblast function, either to promote or suppress immunity, thereby leading to therapies for treating infections, cancer and autoimmune disease.

### Conflict of interest statement

The authors declare that the research was conducted in the absence of any commercial or financial relationships that could be construed as a potential conflict of interest.
